# Is there a relation between stillbirth and low levels of vitamin D in the population? A bi-national follow-up study of vitamin D fortification

**DOI:** 10.1186/s12884-023-05673-8

**Published:** 2023-05-17

**Authors:** Pelle G. Lindqvist, Mika Gissler, Birgitta Essén

**Affiliations:** 1 Clinical Sciences and Education, Obstetrics and Gynecology, Karolinska Institutet, Södersjukhuset, Sjukhusbacken 10, Stockholm, 11883 Sweden; 2 Department of Obstetrics and Gynecology, Södersjukhuset, Stockholm, Sweden; 3 Department of Knowledge Brokers, THL Finnish Institute for Health and Welfare, Helsinki, Finland; 4 Research Centre for Child Psychiatry and Invest Research Flagship, University of Turku, Turku, Finland; 5 Region Stockholm, Academic Primary Health Care Centre, Stockholm, Sweden; 6 Department of Molecular Medicine and Surgery, Karolinska Institutet, Stockholm, Sweden; 7 Department of Women’s and Children’s Health/IMHm, Uppsala University, Uppsala, Sweden; 8 WHO Collaborating Centre On Migration and Health, Uppsala University, Uppsala, Sweden

**Keywords:** Stillbirth, Vitamin D, Fortification, Intrauterine death

## Abstract

**Background:**

Stillbirth has been associated with low plasma vitamin D. Both Sweden and Finland have a high proportion of low plasma vitamin D levels (< 50 nmol/L). We aimed to assess the odds of stillbirth in relation to changes in national vitamin D fortification.

**Methods:**

We surveyed all pregnancies in Finland between 1994 and 2021 (*n* = 1,569,739) and Sweden (*n* = 2,800,730) with live or stillbirth registered in the Medical Birth Registries. The mean incidences before and after changes in the vitamin D food fortification programs in Finland (2003 and 2009) and Sweden (2018) were compared with cross-tabulation with 95% confidence intervals (CI).

**Results:**

In Finland, the stillbirth rate declined from ~ 4.1/1000 prior to 2003, to 3.4/1000 between 2004 and 2009 (odds ratio [OR] 0.87, 95% CI 0.81–0.93), and to 2.8/1000 after 2010 (OR 0.84, 95% CI 0.78–0.91). In Sweden, the stillbirth rate decreased from 3.9/1000 between 2008 and 2017 to 3.2/1000 after 2018 (OR 0.83, 95% CI 0.78–0.89). When the level of the dose-dependent difference in Finland in a large sample with correct temporal associations decreased, it remained steady in Sweden, and vice versa, indicating that the effect may be due to vitamin D. These are observational findings that may not be causal.

**Conclusion:**

Each increment of vitamin D fortification was associated with a 15% drop in stillbirths on a national level. If true, and if fortification reaches the entire population, it may represent a milestone in preventing stillbirths and reducing health inequalities.

## Introduction

Pregnancy risk factors for stillbirth include small-for-gestational age (SGA) fetuses, intrauterine infections, chromosomal aberrations, major malformations, preeclampsia, abruptio placenta, and umbilical cord complications such as vasa previa [1–3]. Maternal characteristics associated with stillbirth are high maternal age, smoking, overweight, and low socioeconomic status [4]. In Sweden and the UK, immigrant background is also associated with an increased risk of stillbirth as compared to native inhabitants [5, 6]. For example, women from the Sub-Saharan region living in Sweden are reported to have a four-fold increased rate of stillbirths compared to the background population, and as a subgroup are characterized by low plasma vitamin D levels [7–10]. A pair of Swedish retrospective analyses of prospective cohorts showed a two-fold increase in birth asphyxia among women with low plasma vitamin D levels. The same study reported a four-fold increase in stillbirths (OR 4.5, 95% CI 1.1–18.2) among women with low plasma vitamin D levels [11, 12].

United Nations Environmental Effects Panel recently stated that additional research is required to assess the health effects of the offspring by sun exposure [13]. Sun exposure is the major contributor to plasma vitamin D levels. However, sun exposure has other non-vitamin D-related effects that are believed to cause an inverse association with hypertension and other cardiovascular diseases [14–17]. Thus, a relationship to low plasma vitamin D might be due to both vitamin D per se, or to some other effects of low sun exposure. Since the Nordic countries are located between 55° and 60° north latitude, their populations experience low UV exposure for a major part of the year. In Europe, a plasma level of 50 nmol/L 25OH vitamin D is regarded as the lower limit of vitamin D sufficiency [18]. A large part (20 to 70%) of the pregnant population has < 50 nmol/L plasma vitamin D levels in both Finland and Sweden [12, 18–20].

Due to low plasma vitamin D levels in the population (mean 48 nmol/L), Finland introduced a voluntary vitamin D food fortification program in 2003. It was recommended that all liquid dairy products, yoghurt, juices, and cereal-based beverages be fortified with 0.5µgr vitamin D per 100 g, and 10µgr/100 g in the case of all fat-based spreads (margarine, etc.) [21]. The recommendation was largely followed. However, due to insufficient vitamin D intake in the population from food (an increase from 3.3 µg/d to 4.6 µg/d) [21], the fortification level was doubled in 2010 to 1.0µgr/L and 20µgr/100gr, respectively [20]. In Finland, with the fortified food intake (8.3 µg/d) and use of vitamin D supplementation (10 µg/d) increased from 28 to 53%, resulting in a vitamin D intake of 16.6 µg/d among women [22]. After 2010 the mean population plasma level of vitamin D had risen from 48 nmol/L to 66 nmol/l, with < 10% of the population having low levels (< 50 nmol/L). In 2018 Sweden implemented a mandatory fortification program of all products produced in Europe (similar to Finland in 2010) [18]. Sweden had previously implemented the mandatory fortification of low-fat milk (3.8 to 5.0 µg/L) and solid margarine (7.5 to 10 µg/100gr) in 2010 [18]. The Swedish recommendation is that there be a 10 µg to 20 µg/d daily intake of vitamin D, depending on sun exposure; the average vitamin D intake is 6 µg/d. Pregnant women who do not consume fortified products or eat fish, or who wear garments covering most of their body, are advised to consult their midwife about vitamin D supplementation [18].

Low sun exposure has been associated with an increased risk of a small-for-gestational age (SGA) pregnancy, the major contributor to stillbirth [23]. In addition, a high body mass index (BMI) is related to lower plasma vitamin D levels; a mendelian randomization study has reported a causal link between high BMI and low plasma vitamin D status [24, 25]. Moreover, low plasma vitamin D levels have been related to lower levels of antibacterial proteins and increased infections [26]. Thus, several of the above mentioned risk factors for stillbirth seem to be associated with low vitamin D levels.

Our aim was to assess whether changes in the national vitamin D fortification programs in Finland and Sweden intended to assure vitamin D sufficiency were related to a reduced incidence of stillbirth.

## Methods

### Study design and data source

We performed a retrospective study of all pregnancies ending in live or stillbirths in Finland (*n* = 1,569,739) and Sweden (*n* = 2,800,730) between 1994 and 2021. The data was taken from the Finnish and Swedish Medical Birth Registries (Approximately 30% of all Swedish births and 5% of all Finnish births are to parents with an immigrant background).

Stillbirth was defined as the birth of a fetus that showed no evidence of viability ≥ 22 + 0 weeks of gestation or ≥ 500 g. In Finland this definition was used for the entire study period, while in Sweden it was used after 2007. (For the years prior to 2007, an old definition of stillbirth was used, i.e., ≥ 28 gestational weeks or, if not known, ≥ 1000 g.) Approximately 15% of all stillbirths in Sweden occur between 22 to 27 weeks of gestation [27]. Therefore, in Table 1 we also present the assumed number of stillbirths using the ≥ 22 week of gestation definition [27]. However, in comparing the Swedish data, only pregnancies after 2007 were included.

**Table 1:** Stillbirths by number of births in Finland and Sweden between 1994 to 2021, percentage smoking, mean maternal age, and BMI > 30

	**1994**	**1995**	**1996**	**1997**	**1998**	**1999**	**2000**	**2001**	**2002**	**2003**	**2004**	**2005**	**2006**	**2007**
**Finland**														
Deliveries (n)	64,142	62,199	59,744	58,345	56,053	56,708	55,855	55,136	54,685	^b^	56,874	56,961	58,156	58,023
Stillbirths (n)	254	302	244	242	238	210	230	211	214		190	185	192	206
Incidence (n/1000)	3.96	4.86	4.08	4.15	4.25	3.70	4.12	3.83	3.91		3.34	3.25	3.30	3.55
Smoking (%)	15.4	15.1	15.2	15.0	15.0	14.8	14.8	15.4	15.6		15.4	14.9	15.1	15.1
Maternal age (y)	29.5	29.7	29.7	29.8	29.9	29.9	29.9	29.9	29.9		30.0	30.0	30.0	30.0
BMI > 30 (%)													10.7	10.8
**Sweden**														
Deliveries (n)	10,9548	100,591	93,270	87,632	84,422	84,722	88,324	89,087	93,596	96,847	99,564	99,357	103,123	10,4445
Stillbirths (n)	352	350	331	351	320	319	342	332	326	332	313	295	308	321
Incidence (n/1000)	3.21	3.48	3.55	4.01	3.79	3.77	3.87	3.73	3.48	3.43	3.14	2.97	2.99	3.07
adj incidence^a^	3.78	4.09	4.18	4.71	4.46	4.43	4.56	4.38	4.10	4.03	3.70	3.49	3.51	3.62
Smoking (%)	18.3	17.1	16.1	14.5	12.7	12.8	12.3	11.2	10.6	9.5	8.8	8.4	7.5	7.2
Maternal age (y)	28.6	28.7	28.9	29.1	29.4	29.5	29.6	29.8	29.9	30.1	30.2	30.3	30.3	30.3
BMI > 30 (%)	7.0	7.7	7.9	8.6	9.2	9.7	10.0	10.5	10.7	10.9	11.2	11.2	11.5	11.9

	**2008**	**2009**	**2010**	**2011**	**2012**	**2013**	**2014**	**2015**	**2016**	**2017**	**2018**	**2019**	**2020**	**2021**
**Finland**														
Deliveries (n)	58,923	59,915	^b^	59,381	59,034	57,724	57,020	55,006	52,861	50,136	47,266	45,285	49,024	49,079
Stillbirths (n)	194	207		163	164	152	166	171	161	145	136	127	122	135
Incidence (n/1000)	3.29	3.45		2.74	2.78	2.63	2.91	3.11	3.05	2.89	2.88	2.80	2.49	2.75
Smoking (%)	15.2	15.7		15.8	16.6	16.0	15.3	14.7	14.2	12.5	11.0	10.7	9.2	7.9
Maternal age (y)	30.1	30.1		30.2	30.3	30.4	30.5	30.6	30.7	30.9	31.0	31.2	31.3	31.6
BMI > 30 (%)	10.9	11.4		12.4	12.6	13.1	12.8	12.9	13.3	14.0	15.4	15.8	16.8	17.9
**Sweden**														
Deliveries (n)	10,6792	108,195	113,454	109,764	110,962	111,517	113,969	115,232	120,110	115,940	^b^	114,564	112,902	112,801
Stillbirths (n)	390	449	430	443	437	427	464	432	446	434		370	354	367
Incidence (n/1000)	3.65	4.15	3.79	4.04	3.94	3.83	4.07	3.75	3.71	3.74		3.23	3.14	3.25
adj incidence^a^														
Smoking (%)	6.9	6.8	6.5	6.3	5.9	5.6	5.6	5.2	4.8	4.6		3.9	3.7	3.2
Maternal age (y)	30.3	30.3	30.3	30.3	30.3	30.3	30.3	30.3	30.3	30.5		30.7	30.9	31.0
BMI > 30 (%)	11.8	11.9	12.6	12.7	13.1	13.0	13.1	13.6	14.1	15.1		15.7	16.3	16.9

Smoking in early pregnancy, mean maternal age, body mass index (BMI) > 30

Finland: BMI data are available from the whole country since 2006

^a^Adjusted for another definition of Stillbirth, i.e., ≥ 28 weeks of gestation instead of ≥ 22 weeks (approximately 15% occur before 28 weeks)

^b^The years of implementation of changed vitamin D fortification are excluded

### Statistical analysis

Excluding the year in which changes were made in the fortification programs, we compared the mean incidence before and after changes in national vitamin D fortification programs (i.e., 1994 to 2002 vs 2004 to 2009, and 2004 to 2009 vs 2011 to 2021 in Finland; and 2007 to 2017 vs 2019 to2021 in Sweden). Cross-tabulations with 95% CI were calculated. Our study is based on publicly available statistical data and no ethical approval or permission to use summary register data is required (Finland Research Ethics Working Party, Swedish Ethics Review Authority).

## Results

**Fig. 1:**
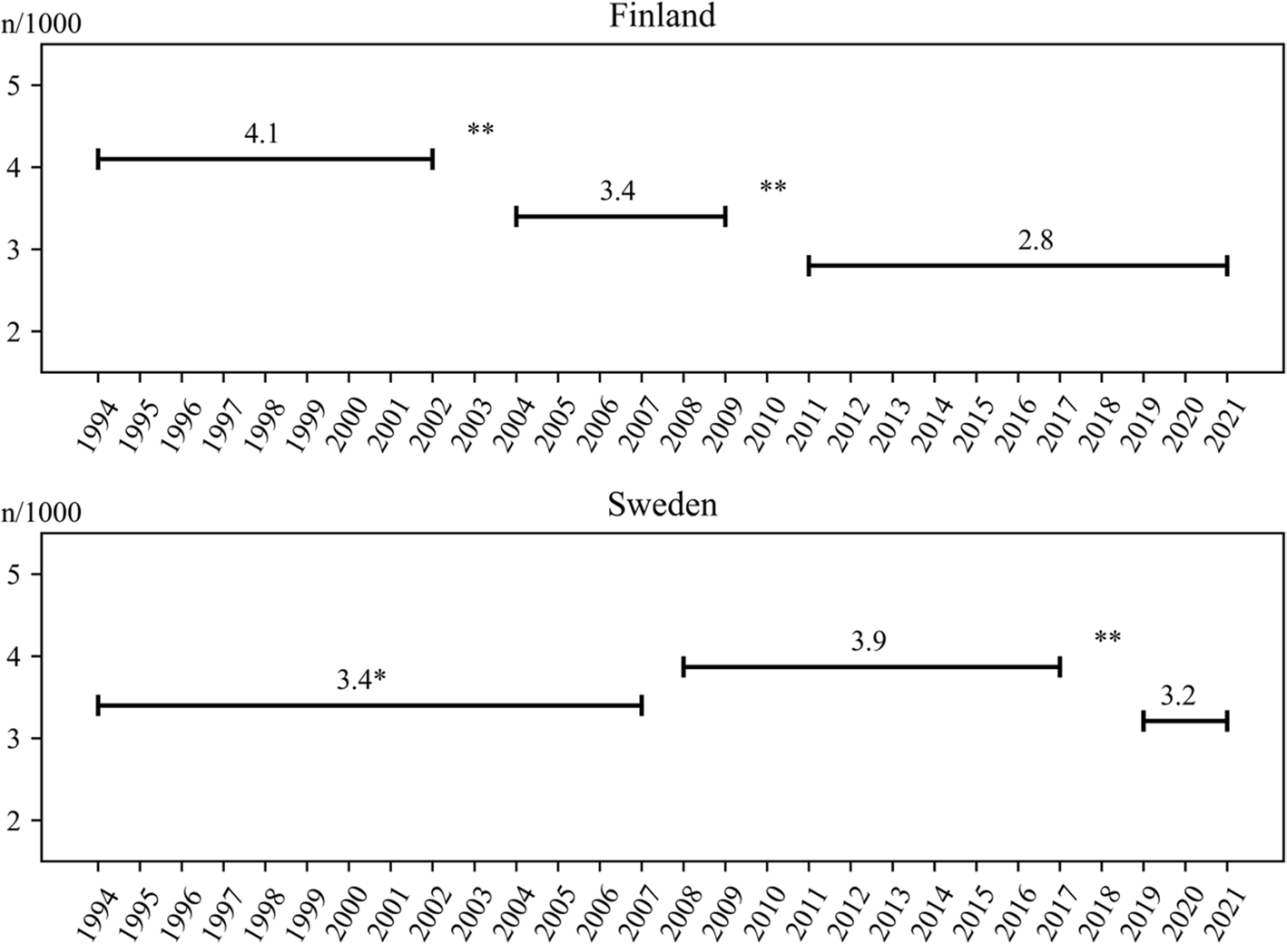
Mean stillbirth rate in Finland (*n* = 1,569,739) and Sweden (*n* = 2,916,899) between 1994 to 2021 by changes in vitamin D fortification. Finland increased fortification 2003 and 2010 and Sweden in 2018, yearly data is given in Table 1. *Definition of stillbirth in Sweden was ≥ 28 weeks of gestation until 2007, which corresponds to ~ 15% lower cases than ≥ 22 weeks. **significance of difference in all three comparisons, *p* < 0.001

In Finland, each increase in vitamin D fortification resulted in a 13% reduced incidence of stillbirth in 2003 (between 1994 to 2002 vs. 2004 to 2009, OR 0.87, 95% CI 0.81–0.93), and a 16% reduced incidence in 2010 (2004 to 2009 vs. 2011 to 2021, OR 0.84, 95% CI 0.78 –0.91) (Fig. 1, Table 1). The stillbirth incidence in Finland decreased from 4.1/1000 (prior to 2003), to 3.4/1000 (2004 to 2009), and 2.8/1000 after 2009, representing a strong dose-dependent reduction in that country (*p* < 0.001). The mean Swedish stillbirth incidence from 2008 to 2017 was 3.9/1000, with a 17% reduction (2008 to 2017 vs. 2019 to 2021, OR 0.83, 95% CI 0.78 –0.89) after the implementation of vitamin D fortification in 2018 (Fig. 1, Table 1); the stillbirth rate dropped to 3.2/1000. All of the above differences are highly significant (*p* < 0.001). In Table 1 we also show the diminishing percentage of smokers in early pregnancy, the increasing maternal age, and the increasing number of women with a BMI ≥ 30.

## Discussion

Finnish observational data indicate a large dose-dependent decreased incidence of stillbirth with each increasing national vitamin D fortification. When the stillbirth rate decreased in Finland, it remained constant in Sweden and vice versa. Thus, a correct temporal connection appeared for an inverse relationship between vitamin D fortification (and a decrease in the prevalence of low plasma vitamin D levels in the population) and stillbirths. The data is in line with the prior study relating low plasma vitamin D levels to increased odds of stillbirth [12]. Although the result was not significant, it was of the same magnitude as the systematic review by Bialy et al. [28]. This indicates the possible effect is associated with vitamin D per se.

### Strengths and limitations

The large study population of > 4 million from two high latitude countries where a considerable portion of the inhabitants have low plasma vitamin D levels is a strength. The large dose-dependent decrease in stillbirth rates (in Finland) and the temporal correlations between changes in the vitamin D fortification programs and stillbirth incidence increase the probability that the observed lower incidence of stillbirth with increasing vitamin D fortification was not a coincidence. A main shortcoming is that we lack individual measurements of plasma vitamin D, but must rely on population level data. A second shortcoming is that we have not determined a plausible mechanism and thus cannot exclude other reasons for the change. The reduced proportion of pregnant smokers has likely decreased the stillbirths, while increasing maternal age and BMI ≥ 30 work in the opposite direction. Improving early detection of severe malformations and chromosomal aberrations that indicate termination of pregnancy should reduce the number of stillbirths. The changes cited above occur gradually. Increasing the identification rate of SGA along with adequate monitoring should also reduce stillbirths. No late pregnancy fetometry screening was implemented in Sweden during the study period [1, 29]. A greater number of inductions at 41 weeks of gestation will reduce the number of stillbirths > 41 weeks. However, since such a practice would take place at the same time routine 41 week fetometry was discontinued, it may increase the number of other severe adverse outcomes in non-identified SGA pregnancies [1, 30–32]. Enhancements in delivery surveillance, such as improved cardiotocography (CTG) guidelines, will have no (or minimal) effect on stillbirth rates because the peripartum stillbirth rate is already low (1/20,000) [33]. Finally, our retrospective design does not have the same strength as a prospective randomized controlled study (RCT), nor might our findings be representative for regions at lower latitudes.

### Possible mechanisms

One hypothesis arises from the fact that muscle strength is dependent on sufficient vitamin D levels [34]. Hence, decreased fetal heart muscle strength might explain the doubled risk of birth asphyxia and emergency cesarean delivery due to fetal distress [11, 12]. The fetal heart might also be more susceptible to increased cardiovascular resistance in growth restriction leading to stillbirth [12]. A large RCT of vitamin D supplementation is almost impossible to perform in the case of a rare event such as a stillbirth. A systemic review of vitamin D supplementation showed a non-significant 25% reduction in stillbirths (relative risk 0.75, 95% CI 0.5–1.12), i.e., corresponding to our findings but still underpowered [28]. A longitudinal study of fetal heart function during deficiency and after sufficiency like the one performed by Herling et al. might address this hypothesis [35]. Greater knowledge might be had by measuring plasma vitamin D levels in women who have, and have not, had stillbirths, and by assessing the relationship between stillbirth and both sun exposure and seasonality. It remains unclear however, what part of pregnancy is most susceptible to low vitamin D levels.

The large US VITAL trial is informative [36]. It measures the effects of five-years of 50 µg/d (2000 IU) of vitamin D supplementation. Almost all subgroups showed an approximately 25 nmol/L increase in plasma vitamin D levels in the supplementation arm, as measured from baseline [36]. Thus, we might estimate that 2000 IU of daily supplementation given to those with insufficient plasma vitamin D levels (25 to 49 nmol/L) would be adequate, while those who are clearly deficient (< 25 nmol/L) might not reach sufficiency. The upper tolerable dose of vitamin D in Europe is 100 µg/d (4000 IU) [18]. In Finland, approximately half the total vitamin D intake is from food fortification and the other half from the recommended supplementation (10 µg/d) [18, 22].

Sweden is the one country in Europe that has had the greatest influx of migrants from low-resource countries per capita over the last decades [37]. In Sweden the recommended intake via food is 10 µg/d vitamin D. Those who eat fortified products receive approximately that amount. However, there is no general, easy to follow recommendation regarding vitamin D supplementation for gravida. There is a recommendation encouraging pregnant women to consult with their midwife at an antenatal care clinic [18]. This may not be adequate because a) the immigrant subgroup commences antenatal care later and makes fewer visits than the general population, and b) the guidelines are not easy to interpret for health personal [38]. In addition, the Swedish fortification program only includes food produced in Europe, whereas non-European food is common in immigrant communities. Fewer dairy products are consumed there due to custom and lactose intolerance, and less fat spreads are used. Thus, a large subgroup of pregnant women with a 3 to fourfold increased risk of stillbirth might not be receiving the optimum vitamin D intake. Hypothetically, the health inequalities and the higher stillbirth rates among immigrant women might at least partly be due to differences in plasma vitamin D [39]. In terms of equal health there might be a socio-economic benefit of relying more on fortification and less on supplementation or sun exposure. On the other hand, sun exposure seems to have additional non-vitamin D dependent advantages, such as lower risk of type 2 diabetes mellitus, hypertension, venous thromboembolism, and other cardiovascular diseases (CVD), non-CVD/non-cancer, and cancer mortality [17, 40–42]. The fortification of cooking oil or flour might be alternatives to reach immigrant women. Although national food agencies in Finland and Sweden may have made a major public health contribution in lowering the incidence of stillbirth by their vitamin D fortification/ supplementation programs, it would be important to assess whether those programs have had their intended effect and reaching all women.

### Modifiable risk factors for stillbirth

Approximately half of all pregnancies ending in stillbirth are SGA [1, 43]. Identifying SGA in combination with an umbilical artery Doppler surveillance program has been reported to bring about a 5- to sixfold reduction in the risk of stillbirth (SGA has a population-attributable risk of ~ 11%) [1, 43, 44]. A Cochran review has advised against late ultrasound fetometry screening [29]. However, this Cochrane review did not include stillbirth without congenital abnormalities as an outcome [29]. Previously, smoking was the major population-attributable risk factor for stillbirth. However, smoking cessation programs have been effective in reducing smoking in Sweden from 18% in early 1994 to < 4% today (Table 1) [37]. On the other hand, while the proportion of smokers is decreasing, BMI is increasing (Table 1) [45]. Obesity is also a modifiable major risk factor for stillbirth [46]. There is an inverse correlation between BMI and plasma vitamin D levels, and, as cited earlier, a mendelian randomization study has reported a causal link between high BMI and low plasma vitamin D status [4, 24, 25]. If our hypothesis is true, low plasma vitamin D levels might also be a high ranking risk factor for stillbirth.

## Conclusion

Our observational data of > 4 million pregnancies show that in two Northern countries with widespread low plasma vitamin D levels (< 50 nmol/l), each increment of national vitamin D food fortification was associated with an approximately 15% reduction in stillbirths. If a causal relationship can be established, a milestone would be achieved in preventing stillbirths in Northern countries. Food fortification that reaches the entire population might be a step towards equal reproductive health, independent of socioeconomic and ethnic background.

## Data Availability

All data analyzed during this study are included in this published article.

## Abbreviations

BMI: Body mass index
CI: Confidence interval
CVD: Cardiovascular diseases
CTG: Cardiotocography
OR: Odds ratio
SGA: Small-for-gestational age
UV: Ultraviolet
